# LEF1 mediates osteoarthritis progression through circRNF121/miR-665/MYD88 axis via NF-кB signaling pathway

**DOI:** 10.1038/s41419-020-02769-3

**Published:** 2020-07-30

**Authors:** Tianfu Wang, Zhiyu Hao, Changcheng Liu, Lebin Yuan, Li Li, Menghong Yin, Qing Li, Zhiming Qi, Zi Wang

**Affiliations:** 1 Department of Sports Medicine, Dalian Municipal Central Hospital, Dalian, 116033 Liaoning Province China; 2 Department of Spinal Surgery, The Second Hospital of Dalian Medical University, Dalian, 116033 Liaoning Province China; 3 Department of Medical Imageology, Dalian Medical University, Dalian, 116044 Liaoning Province China

**Keywords:** Mechanisms of disease, Inflammation, miRNAs

## Abstract

Osteoarthritis (OA) is a joint disease that causes great pain to patients and imposes a tremendous burden on the world’s medical resources. Regulatory noncoding RNAs, including circular RNAs (circRNAs) and microRNAs (miRNAs), play an important role in OA progression. Here, we identified differential expression of transcription factor LEF1 that increased circRNA circRNF121 levels in normal and OA cartilage tissues. The expression of LEF1 and circRNF121 was positively associated with Mankin’s scores. Alteration of circRNF121 mediated the degradation of extracellular mechanisms (ECM), apoptosis, and proliferation of chondrocytes. MiR-665 was identified as a direct regulatory target of circRNF121 and MYD88. Functional analysis showed that circRNF121 and MYD88 modulated ECM degradation, apoptosis, and proliferation of chondrocytes, which could be reversed by miR-665. MYD88 regulated the activity of the NF-кB signaling pathway by circRNF121 via sponging miR-665. Collectively, these data indicated that LEF1 impacted OA progression by modulating the circRNF121/miR-665/MYD88 axis via NF-кB pathway. Our research proposed a new molecular mechanism for the development of OA, and provided a prospective therapeutic target for OA.

## Introduction

Osteoarthritis (OA) is a disease associated with biomechanical and biological factors. OA includes changes in the mechanical environment around the joints, apoptosis, and damage of chondrocytes, as well as changes in the components of the cartilage matrix. The pathological manifestation of OA is a process of biologic reconstruction, which is characterized by degenerative lesions of articular cartilage (AC) hyperplasia and hypertrophy^1,2^. OA has a marked impact on the quality of life of patients. At this stage, nonsurgical treatments, such as hyaluronic acid (HA) injection, platelet-rich plasma (PRP) injection, and stem cell therapy for OA are particularly important. However, due to the lack of knowledge of the molecular mechanism, the treatment on OA is not always effective. Therefore, it is urgent to clarify the potential molecular mechanism during OA progression for the development of therapy.

Lymphoid enhancer-binding factor 1 (LEF1) is a transcription factor involved in many diseases^3–6^. It belongs to the high-mobility group family that regulates gene expression by inducing structural alteration in the DNA helix^7^. Previous study indicates that IL-1β mediates LEF1 expression, which facilitates MMP-13 gene expression via a DNA looping mechanism^8^. Jinan et al. demonstrate that SIRT1 represses MMP-13 in human OA chondrocytes mediated by LEF1. Nevertheless, the potential molecular mechanism of LEF1 on OA progression has not been fully elucidated.

Endogenous noncoding RNA (ncRNA), such as circular RNA (circRNA) and microRNAs (miRNAs), has been widely concerned. CircRNA forms a complete circular structure by connecting the 3′ and 5′ terminals together through exon or intron cyclization. CircRNAs play important roles in life activities, such as epigenetic modulation, cell cycle, and differentiation regulation^9–12^. miRNAs are single-stranded RNA molecules with approximately 22 nucleotides in length. They are widely involved in the regulation of gene expression to influence cell function^13,14^. Shen et al. report that overexpression of circSERPINE2 functions as competing endogenous RNA (ceRNA) of miR1271 to regulate anabolism of the extracellular matrix (ECM) in OA progression^15^. However, the underlying mechanism of circRNF121 and miR-665 on OA progression has not been elucidated.

NF-κB family has essential roles in a wide range of biological processes, including cell survival, proliferation, differentiation, apoptosis, aging, inflammation, and immune responses^16–20^. MYD88 is the canonical adaptor for inflammatory pathway member of Toll-like receptor (TLR) and interleukin-1 (IL-1) receptor families, which modulate the NF-κB signaling pathway^21–23^. However, it is still essential to clarify the potential mechanisms of MYD88 and NF-кB pathway during OA.

The purpose of this study is to explore the underlying mechanism of OA processes involved in LEF1-mediated NF-кB pathway regulation by circRNF121/miR-665/ MYD88 axis.

## Materials and methods

### Bioinformatics analysis

The Gene Expression Omnibus (GEO, http://www.ncbi.nlm.nih.gov/geo/) was searched for OA datasets. Microarray data of mRNAs were collected from GEO datasets (GSE114007) via the GPL11154 platform. Raw files of microarray data were downloaded and normalized using a robust multiarray averaging method with ‘affy’ and ‘simpleaffy’ packages of ‘R’ software (www.R-project.org/). The processed gene expression matrix is provided in Supplementary Table S1. The ‘pheatmap’ and ‘ggplot2’ packages of ‘R’ were applied to visualize the data. The enrichment analyses were calculated by datasets of GO (geneontology.org)^24^. The data of ChIP-seq were collected from Cistrome Data Browser (cistrome.org/db/#/). After searching for the keyword ‘LEF1’, data were analyzed in the current study (53651_None_LEF1, 63332_R562_LEF1, 63333_R562_LEF1).

### Clinical samples

The OA tissue of patients who underwent total knee arthroplasty (*n* = 30, age 63.7 ± 6.8 years) was gathered from December 2017 to March 2018. At the same time, we collected cartilage tissue from the amputees without rheumatoid arthritis or OA (*n* = 5, age 39.8 ± 5.3 years). All the tissues were taken from medial tibial plateau. The diagnosis of these people was according to the European League Against Rheumatism/American College of Rheumatology classification^25^. After surgery, tissue samples were stored with liquid nitrogen before the experiment. Part tissues from normal patients were directly digested for cell culture. This study was approved by the Research Ethics Committee of Dalian Central Hospital (DLMCH-2017-0014). All participants read and signed the informed consents.

### Real-time PCR analysis

The total RNA was isolated by RNeasy Mini Kit (74106, Qiagen, Valencia, CA) from human cartilage tissues and cultured chondrocytes. The cDNA was synthesized by QuantiTect Reverse Transcription Kit (205314, Qiagen, Valencia, CA). The relevant primers of the current study are listed in Supplementary Table S2. Quantitative real-time PCR (q-PCR) was done by using QuantiFast SYBR Green PCR Kit (204056, Qiagen, Valencia, CA). Relative RNA expression was calculated using the 2^−ΔΔCT^ method with normalization to U6 small nuclear RNA or GAPDH. Each experiment was performed in triplicate.

### OA cell culture

The chondrocytes were extracted from normal patients’ cartilage tissue as previously described. Briefly, cartilage tissue was cut into as small pieces as possible and then digested with 0.1% trypsin (15400054, Gibco Company, USA) for 30 min. Thereafter, it was digested with a Type II collagenase (17101015, Gibco Company, USA) in Dulbecco’s modified Eagle’s medium (DMEM, 11965092, Gibco Company, USA) at 37 °C for 10 h. Undigested tissue was filtered using a 40-nm strainer. The chondrocytes were then cultured in DMEM containing 10% fetal bovine serum (30044333, Gibco Company, USA). The second and third passage cultured chondrocytes were used for the experiment.

### Cell transfection

The cDNA of LEF1, circRNF121, and MYD88 was cloned into the multiple cloning site of the pcDNA3.1 vector (Invitrogen, USA). MiR-665 mimic, negative control oligonucleotides (miR-NC), miR-665 inhibitor, negative control oligonucleotide (NC inhibitor), small-interfering RNA of LEF1, circRNF121, or MYD88 (siLEF1, sicirc-1, sicirc-2, sicirc-3, and siMYD88), and scramble siRNA of LEF1 circRNF121 or MYD88 (siSCR) were purchased from RiboBio (Guangzhou, China). Lipofectamine 3000 (Invitrogen, Carlsbad, CA, USA) was used for the transfection assay in a 6-well plate with seeded chondrocytes. Q-PCR was used to detect the expression of mRNA. Transfection efficiency was detected by fluorescence microscope (OLYPAS, Japan). After 48 h, chondrocytes were stimulated with IL-1β (10 ng/ml) for 24 h and used for further analysis.

### Chromatin immunoprecipitation (ChIP)

Chip assay was performed using the EZ-Magna ChIP kit (17-10086, EMD Millipore, GER). Human chondrocytes were fixed with 4% paraformaldehyde and incubated with glycine for 10 min to generate DNA–protein cross-links. Then, the cells were lysed with Cell Lysis Buffer and Nuclear Lysis Buffer and sonicated to generate chromatin fragments of 400–800 bp. The lysates were immunoprecipitated with Magnetic Protein A Beads conjugated with LEF1 antibody (ab137872, Abcam, Cambridge, UK) or IgG. Finally, the precipitated DNA was analyzed by PCR.

### Western blot assay

Cytoplasmic protein and nuclear protein were extracted using a Nuclear and Cytoplasmic Extraction Reagents kit (78835, Thermo Fisher Scientific, USA). The protein concentration was measured by the BCA Assay Kit (23223, Thermo Scientific, USA). The protein was electrophoresed with 10% sodium dodecyl sulfate (SDS) polyacrylamide gel electrophoresis, and subsequently transferred to the polyvinylidene difluoride (PVDF) membranes (Millipore, Bedford, MA, USA). Specific primary antibodies (ab181602, ab32535, ab39012, ab41037, ab188570, ab186414, ab32042, ab32561, ab219413, ab76429, ab133462, ab32536, ab32511, Abcam, Cambridge, UK) (PA5-61136, Invitrogen, USA) were co-incubated with the membrane overnight at 4 °C. The membranes were rewarmed for 2 h, and then incubated with anti-rabbit IgG (ab97051, Abcam, Cambridge, UK) at 37 °C for 2 h. ECL Western blot kit (32209, Thermo Fisher Scientific, USA) was used to detect the bands. GAPDH and Lamin B were used as controls.

### Immunofluorescence (IF) staining

The chondrocytes were cultured in a 12-well plate and stimulated with IL-1β for 24 h. Primitively, cells were fixed with 4% paraformaldehyde for 20 min. Subsequently, the cells were treated with 0.2% Triton X-100 for 3 min, and blocked with 5% bovine serum albumin for 1 h. The cells were incubated with primary antibody (Abcam, Cambridge, UK) overnight at 4°C. The chondrocytes were washed with PBS and incubated with goat anti-rabbit IgG (SA00013-4, SA00013-2, Proteintech, China) for 1 h at room temperature. Last, DAPI(4′,6-diamidino-2-phenylindole) (c0065, Solarbio, China) was used for nuclear staining. The fluorescence was observed with a fluorescence microscope (OLYPAS, Japan).

### CCK-8 assay

Cell Counting Kit-8 (CCK-8) (CK04-500T, Solarbio, China) was used to assess the proliferative capability of chondrocytes. In brief, 48 h after transfection, 10 μL of CCK-8 solution was added to each well and incubated for 4 h. The absorbance at 450 nm was measured using a microplate reader (Model 680, Bio-Rad, USA).

### Flow cytometry

PE Annexin V Apoptosis Detection Kit I (559763, BD Pharmingen, USA) was used to determine the apoptotic rate of treated chondrocytes. Subsequently, the results were analyzed with the fluorescence-activated cell sorting (FACS) flow cytometer (BD Biosciences, USA). Each experiment was performed in triplicate.

### Dual-luciferase reporter gene assay

The pmirGLO Dual-Luciferase miRNA Target Expression Vector was obtained from GenePharma (China). The amplified DNA sequences were cloned to the pmirGLO reporter plasmid to form wild- type MYD88 3′-UTR (WT) and mutated MYD88 3′-UTR (MUT) luciferase vectors. The pmiRGLO luciferase reporter vectors of the circRNF121 were constructed as above. Human chondrocytes were plated (5 × 10^4^ cells per well) in 24-well plates overnight. Lipofectamine 3000 (Invitrogen, Carlsbad, CA, USA) was used to transfect the chondrocytes with plasmid and miR-665 mimic or the control. The luciferase activity was measured by the dual-luciferase reporter gene assay system (E1910, Promega, USA) after 48 h. Data were shown as the mean value ± SD, and each experiment was performed in triplicate.

### RNA immunoprecipitation (RIP) assay

Magna RIPTM RNA Binding Protein Immunoprecipitation Kit (17-700, Millipore, USA) was used to conduct the RIP assay. The endogenous miR-665, combined with circRNF121, was pulled down. Briefly, cultured chondrocytes were collected and resuspended in RIPA buffer (R0020, Solarbio, China). Then the cell extracts were incubated with RIP buffer containing magnetic beads conjugated with human anti-Ago2 antibody (07-590, Millipore, USA) or mouse IgG (CBL610, Millipore, USA) negative control overnight at 4 °C. The next morning, proteinase K was used to co-incubate with the magnetic beads after washing three times. Subsequently, total RNAs were isolated from the extracts using the TRIzol reagent. The relative enrichment of circRNF121 and miR-665 was determined by RT-q-PCR analysis.

### Fluorescence in situ hybridization (FISH) assays

The FISH assays were done by FISH kit (F03402, GenePharma, China). In brief, the probes specific to circRNF121 and miR-665 were added to human chondrocytes and then pre-denatured at 78 °C for 5 min. Then, hybridization was carried out at 42 °C overnight. DAPI (c0065, Solarbio, China) was used to counterstain the nuclei with 20 min in darkness. The sample was scanned and photographed under Olympus microscope.

### Rat model of OA

Animal experiments were performed according to the Guidelines for Animal Experimentation of Dalian Medical University, and the rats were purchased from the Experimental Animal Center of Dalian Medical University. The ethics approval permit number was SYXK-2018-0007. One day before operation, all the rats were randomly divided into 7 groups (Control, DMM, DMM + miR-NC, DMM + miR-665, DMM + vector, DMM + circRNF121, and DMM + MYD88, *n* = 12 per group) after stratification according to body mass. Experimental OA in 10-week-old male SD rats was induced by medial collateral ligament transection and DMM as described previously^26^. MiR-NC, miR-665 agomir (50 μM), and lentivirus (1 × 10^9^ PFU, 20 μl) expressing MYD88 or circRNF121 were injected into the knee joints of recipient rats 1 week after the surgery (20 μL per joint per rat twice a week for 4 weeks). Eight weeks after surgery, rats were killed, and knee joints were harvested. The medial femoral condyles were used for subsequent experiments.

### Histology and immunohistochemical (IHC) staining

Human cartilage tissues and rat knee joints were fixed in 4% paraformaldehyde for paraffin. Tissue was decalcified using EDTA decalcification solution for 2 months. The section was pretreated with drying, deparaffining, and rehydrating. Then the tissues were cut into 4-μm slices. For IHC staining, the sections were incubated with appropriate primary antibodies overnight at 4 °C after dewaxing in xylene. The next day, the slices were incubated with secondary streptavidin–horseradish peroxidase-conjugated antibody at room temperature for 1 h. Finally, the IHC signals were visualized using 3, 3′-diaminobenzidine (DAB) (ZLI9018, ZSGBBIO, China). Safranin-O and fast green-stained (G1371, Solarbio, China) sections were used to evaluate matrix proteoglycan and the overall joint morphology.

### Co-immunoprecipitation (Co-IP) assay

Co-immunoprecipitation was largely carried out as described previously. Briefly, the cultured chondrocytes were lysed and collected the lysates. The protein concentration was measured by the BCA Assay Kit (23223, Thermo Scientific, USA). Protein A/G Magnetic Beads were used to preclear lysates for 1 h at 4 °C with rotation. The A/G Magnetic Beads were removed and then followed by incubation with the immunoprecipitation antibody overnight at 4 °C with rotation. The next morning, the lysates were added to the preblocked A/G magnetic beads and incubated at room temperature with rotation for 1 h. The beads were washed and eluted the immunoprecipitated proteins in SDS-PAGE sample buffer. The bands were detected using the ECL Western blot kit (32209, Thermo Fisher Scientific, USA).

### Statistical analysis

SPSS 17.0 software was used to analyze the data. Data were presented as means ± standard deviation (SD). Student’s *t* test was used to compare the significant difference of two groups. SD represented the variation of data values. The one-way analysis of variance (ANOVA) was used to determine the significant difference of multiple groups. Pearson correlation coefficient was used to identify the correlation. Each experiment was carried out thrice at least. Statistical significance was defined as *P* value < 0.05.

## Results

### The expressional profiles of mRNAs in cartilage tissues

**Fig. 1:**
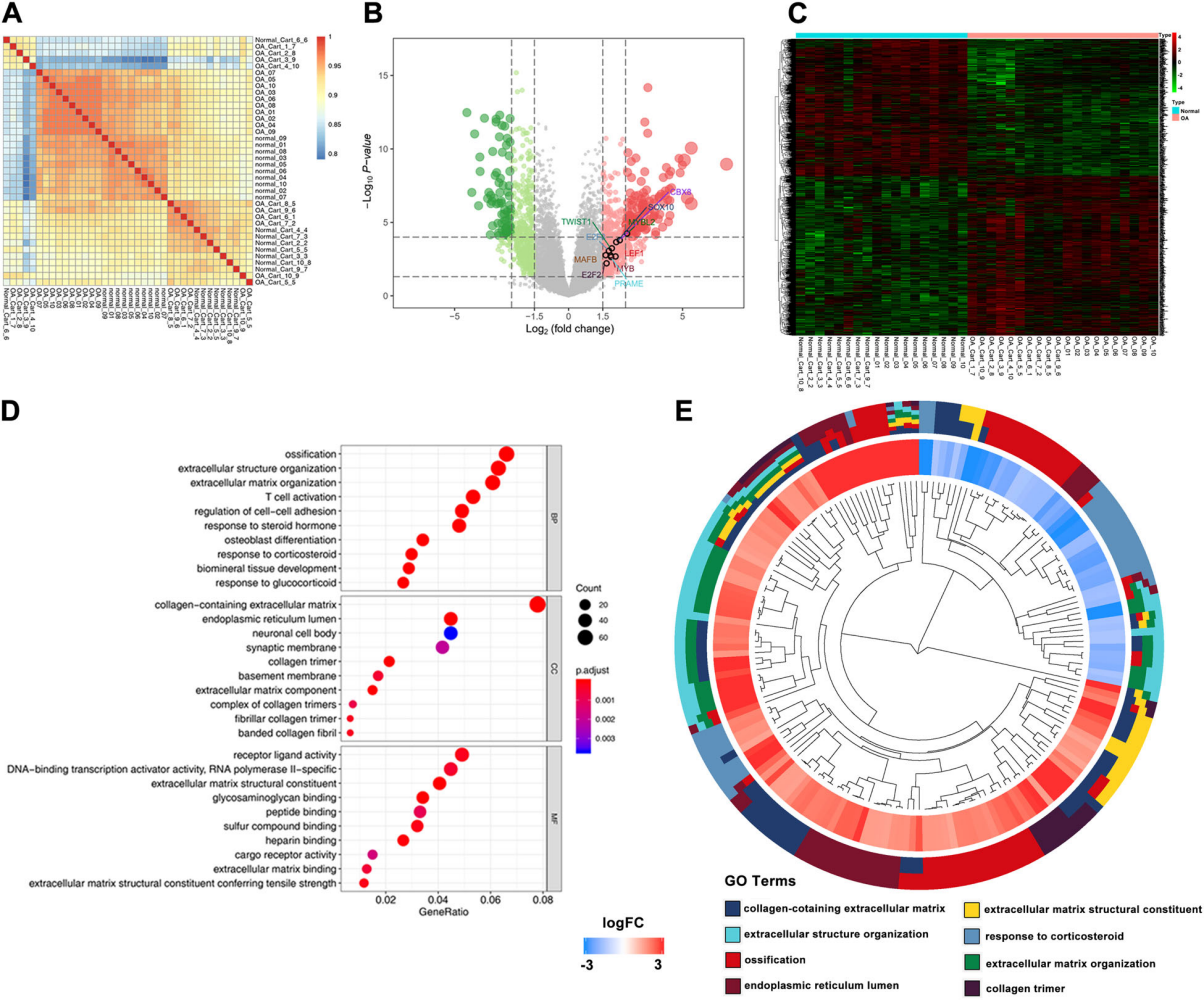
The expressional profiles of mRNAs in cartilage tissues. **a** Correlation of groups of the expression matrix was explored by ‘pheatmap’ package. **b** Volcano plots were constructed using fold change and p values. **c** Heatmap of all differentially expressed mRNAs between OA and normal cartilage samples was shown. **d**, **e** GO analysis between the OA and normal group was shown.

To assess the quality of the data, the correlation between the samples in the expression matrix was analyzed (Fig. 1a). We next visualized the processed circRNA expression matrix. The volcano plots showed varied circRNA expression between the OA and normal cartilage samples. Transcription factors with LogFC > 1.5 were highlighted in the plots (Fig. 1b). The heatmap revealed the mRNA expression in all the samples (Fig. 1c). Furthermore, GO analysis showed that there were many differences in cellular component (CC), molecular function (MF), and biological process (BP) between the OA and normal group (Fig. 1d). The significant differences were related to extracellular mechanisms and collagen expression (Fig. 1e).

### Altered LEF1 modulates the expression of circRNF121 in OA

**Fig. 2:**
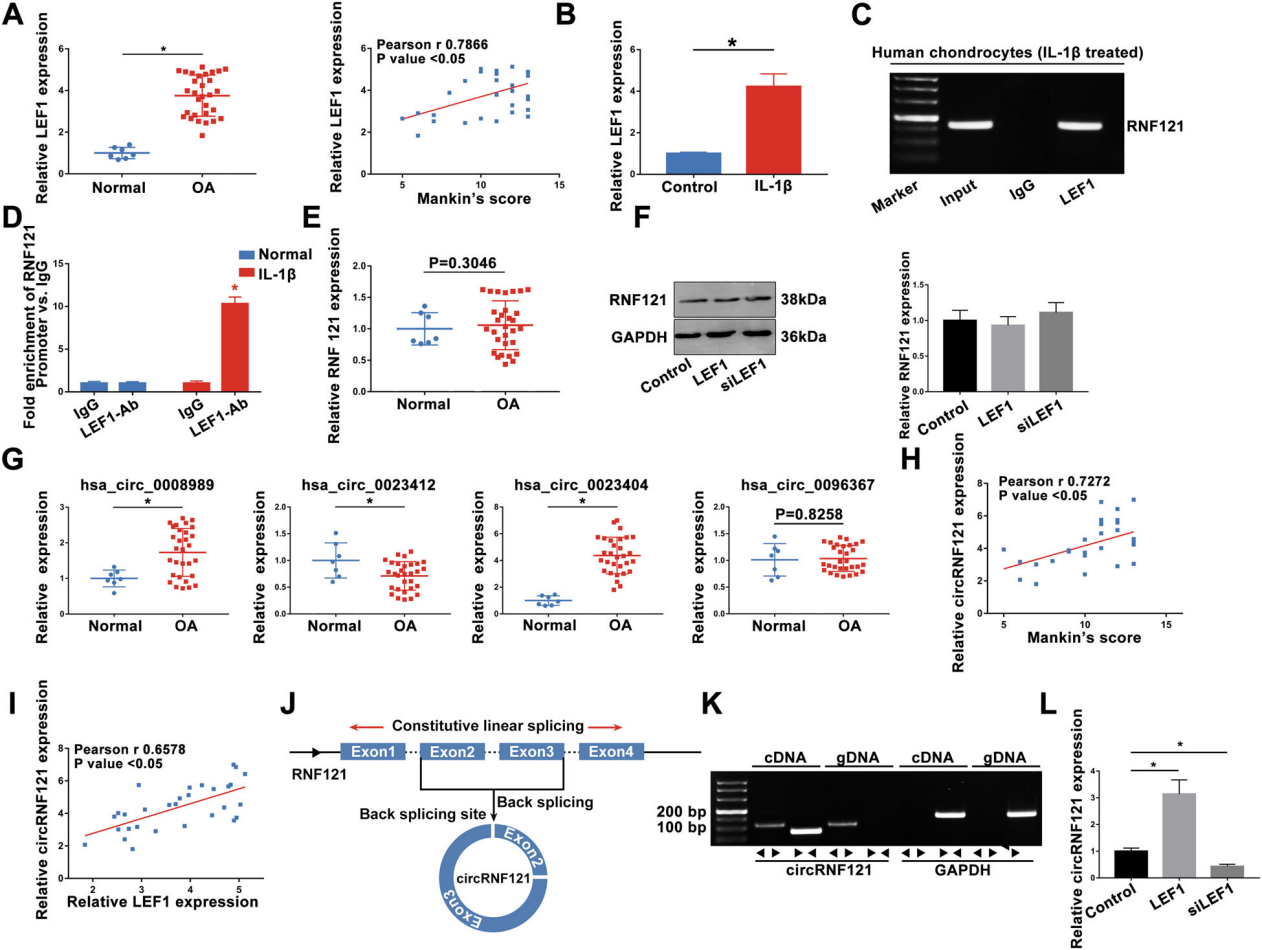
Altered LEF1 modulates the expression of circRNF121 in OA. **a** The expression of LEF1 was identified in OA and healthy human cartilage tissues by q-PCR. **b** The LEF1 expression in chondrocytes treated with IL-1β was detected by q-PCR. **c**, **d** ChIP analysis of human chondrocytes treated with IL-1β using specific antibodies against LEF1 was shown. **e** Expression of RNF121 between OA and normal groups was identified by q-PCR. **f** RNF121 expression in LEF1-altered expressing chondrocytes was detected by western blot. **g** circRNA levels were identified by q-PCR. **h** CircRNF121 level was positively correlated with the modified Mankin’s scores. **i** Correlation analysis between the expression levels of LEF1 and circRNF121 was identified. **j** Exon2 and Exon3 region of RNF121 pre-mRNA was back- spliced and formed circRNF121. **k** CircRNF121 was amplified by divergent primers in cDNA but not in gDNA. GAPDH was used as a negative control. **l** Expression of circRNF121 was detected by q-PCR after transfection. Data were means ± SD of three independent assays (*P < 0.05).

According to the bioinformatics analyses, we hypothesized that LEF1 regulated the progression of osteoarthritis. In the next experiment, a higher level of LEF1 was determined in OA cartilage tissues than that in normal tissues, which was positively correlated with the modified Mankin’s scores (Fig. 2a). The expression of LEF1 was significantly increased after treatment with IL-1β (Fig. 2b). As a transcription factor, LEF1 could active the translation by combining with the promoter region. High-quality Chip-seq data in the Cistrome Data Browser (Supplementary Fig. S1) showed that LEF1 might combine with the promoter region of RNF121. ChIP analysis of human chondrocytes treated with IL-1β using specific antibodies against LEF1 showed occupancy of LEF1 on the RNF121 promoter (Fig. 2c, d). Though LEF1 enhanced RNF121 promoter activity, there was no significant difference in the expression of RNF121 between OA and normal groups in the expression matrix and q-PCR results (Fig. 2e). Altered expression of LEF1 did not alter RNF121 expression in chondrocytes (Fig. 2f). Therefore, circRNAs generated from RNF121 could be concerned to play an important role in OA progression. circRNAs of RNF121 were obtained from circBase and explored (Fig. 2g). Then, we focused on the has_circ_0023404 (circRNF121) in the present study. Interestingly, the expression of circRNF121 was positively correlated with the modified Mankin’s scores (Fig. 2h). Furthermore, circRNF121 expression was positively correlated with the LEF1 expression in the OA cartilage tissues (Fig. 2i). Exon2 and Exon3 region of RNF121 pre-mRNA was back-spliced and formed circRNF121 (Fig. 2j). CircRNF121 was amplified by divergent primers in cDNA but not in gDNA (Fig. 2k). LEF1 regulated the expression of circRNF121 in human chondrocytes (Fig. 2l).

### CircRNF121 mediates OA progression

**Fig. 3:**
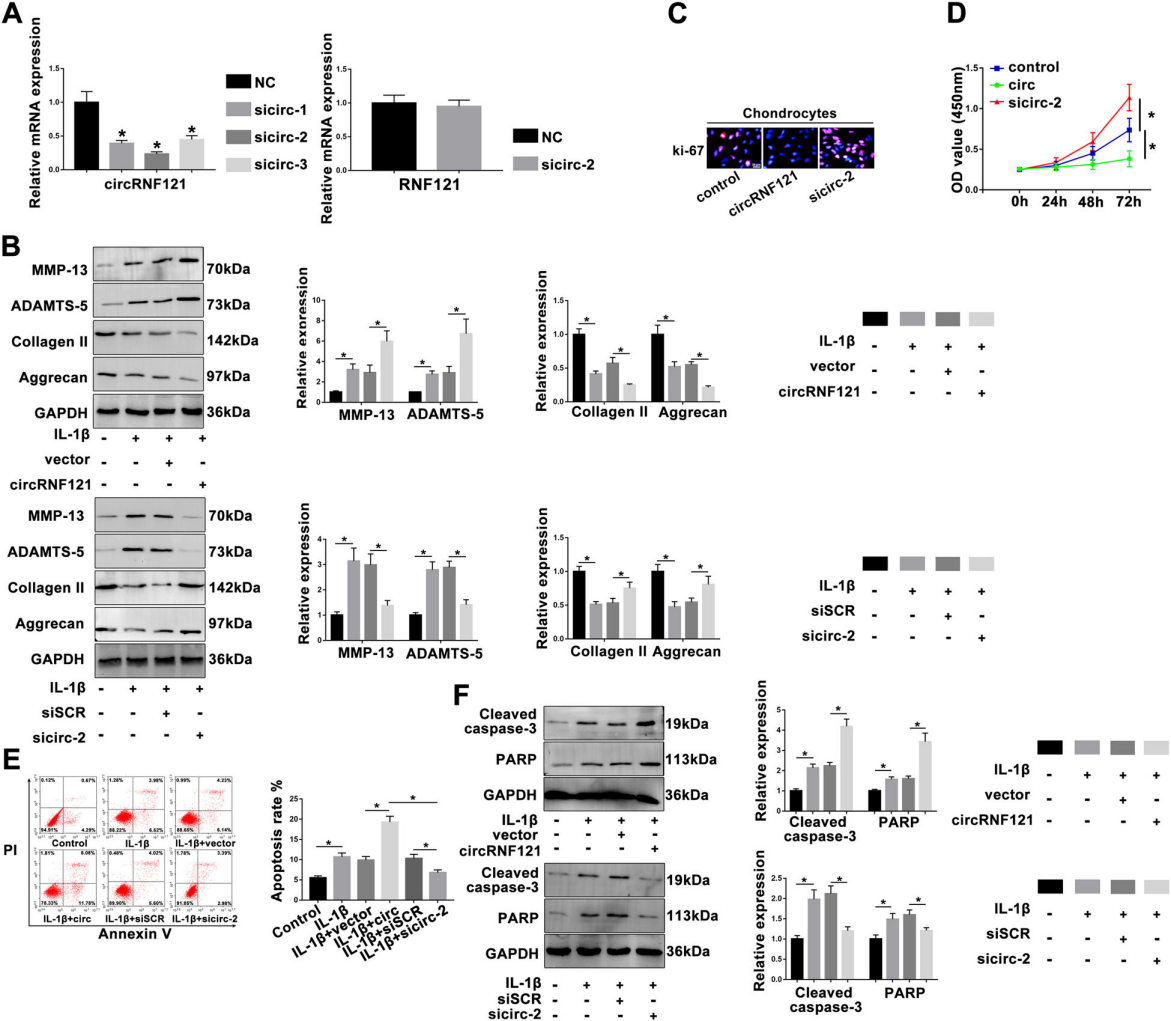
CircRNF121 mediates OA progression. **a** The expression of circRNF121 was identified after transfection of various sicircRNAs by q-PCR. The RNF121 expression was detected by q-PCR in treated chondrocytes. **b** Representative proteins, MMP-13, ADAMTS-5, type II collagen, and aggrecan were analyzed in treated chondrocytes by western blot. **c**, **d** Ki-67 immunofluorescence staining and CCK-8 proliferation assay were used to identify the proliferative capability of chondrocytes after transfecting with circRNF121 or sicircRNA-2. **e** Apoptosis of chondrocytes was evaluated by flow cytometry. Viable and nonviable apoptotic cells are regarded as apoptotic cells. **f** Cleaved caspase-3 and PARP levels were measured by western blot. Data were means ± SD of three independent assays (*P < 0.05).

CircRNF121 small-interfering RNA was transfected to assess circRNF121 expression. Knockdown of circRNF121 expression had no effect on RNF121 mRNA level (Fig. 3a). The chondrocytes were treated with IL-1β (10 ng/ml) for 24 h after transfection, and the associated protein levels of chondrocyte (MMP-13, ADAMTS-5, type II collagen, and aggrecan) were investigated. As shown in Fig. 3b, circRNF121 promoted the expression of MMP-13 and ADAMTS-5, while it inhibited the expression of the main cartilaginous composition of ECM, type II collagen, and aggrecan. The ki-67 immunofluorescence staining and CCK-8 assay indicated that circRNF121 inhibited chondrocyte proliferation. Conversely, inhibition of circRNF121 promoted the proliferation of chondrocytes (Fig. 3c, d). Chondrocytes transfected with circRNF121 showed higher expression of apoptosis-related proteins (cleaved caspase-3 and PARP) than that in the vector group, while chondrocytes transfected with sicirc-2 showed a decreased tendency (Fig. 3f). We further determined the apoptotic rate by flow cytometry (Fig. 3e). These results indicated that abnormal circRNF121 could mediate OA progression by regulating ECM degradation, chondrocyte proliferation, and apoptosis.

### CircRNF121 functions as a sponge of miR-665 and indirectly mediates MYD88 level

**Fig. 4:**
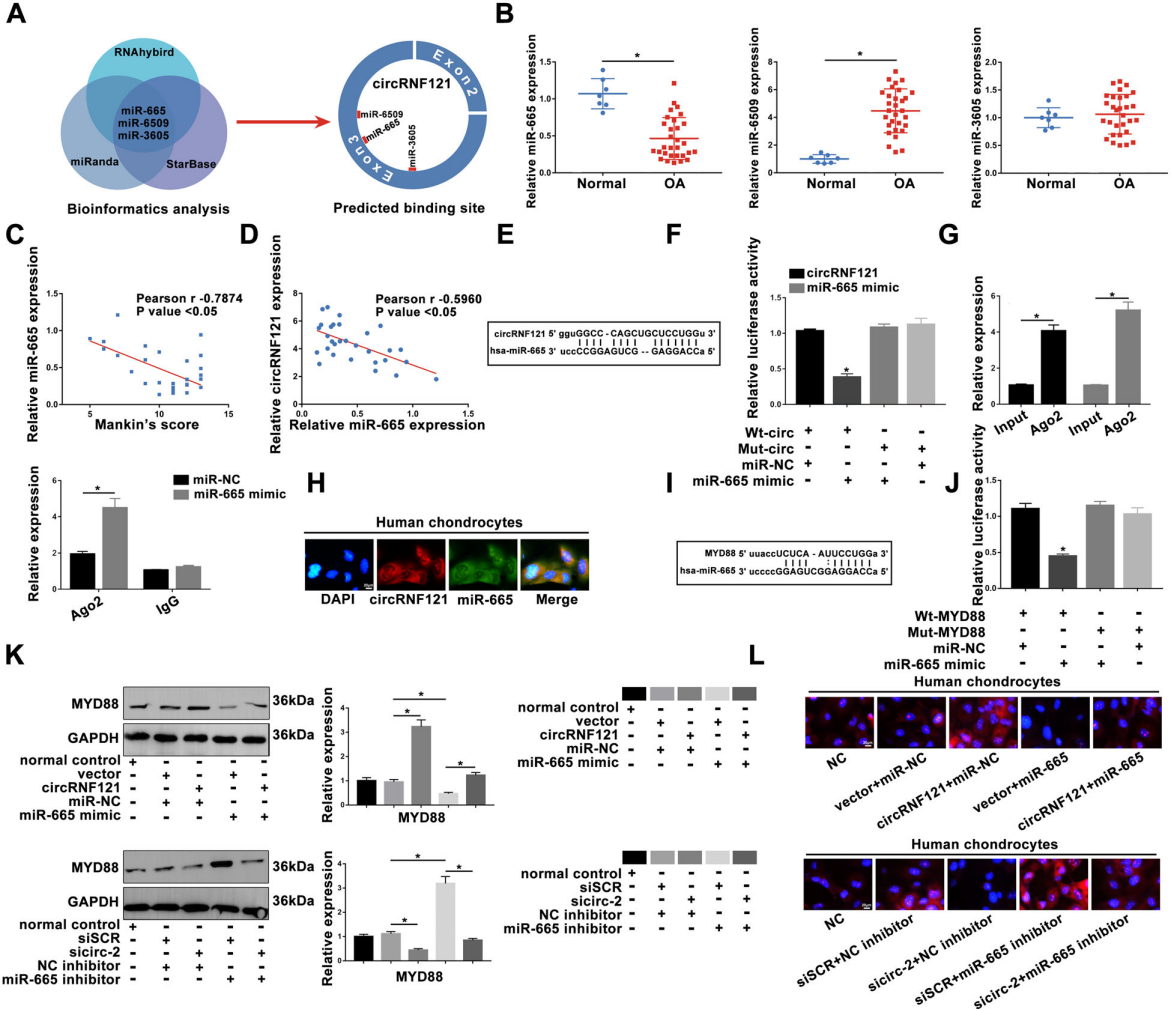
CircRNF121 functions as a sponge of miR-665 and indirectly mediates MYD88 level. a, **b** Three miRNAs were selected and analyzed by q-PCR. **c** The expression of miR-665 was negatively correlated with the modified Mankin’s scores. **d** The correlation analysis of the expressions of circRNF121 and miR-665 was shown. **e** The predicted binding sites between circRNF121 and miR-665 were presented. **f** The dual-reporter luciferase assay confirmed that circRNF121 was the direct target of miR-665. **g** RNA immunoprecipitation was performed in chondrocytes transfected with miR-NC and miR-665 mimic. CircRNF121 expression was detected by using qRT-PCR. RNA levels were presented as fold enrichment in Ago2 relative to IgG immunoprecipitates. **h** The localization of RNF121 and miR-665 was detected by FISH assay in human chondrocytes. **i** The predicted binding sites between MYD88 and miR-665 were shown. **j** The decreased luciferase was also shown to determine the direct binding of MYD88 and miR-665. **k**, **l** MYD88 expression was determined by western blot and IF staining in treated chondrocytes. Data were means ± SD of three independent assays (*P < 0.05).

To explore the underlying mechanisms of circRNF121 on OA progression, three online bioinformatics analysis were used to predict potential miRNAs that might target the circRNF121. Three correlated miRNAs, miR-665, miR-6509, and miR-3605 (Fig. 4a), were found, and the most significant miR-665 was selected for future analysis (Fig. 4b). Interestingly, the level of miR-665 had a negatively correlation with modified Mankin’s scores (Fig. 4c). Furthermore, the level of miR-665 was negatively correlated with circRNF121 (Fig. 4d). As shown in Fig. 4e, f, circRNF121 was a direct target of miR-665 in chondrocytes by dual-luciferase reporter assay. An anti-Argonaute 2 (Ago2) RIP assay verified the endogenous interaction between circRNF121 and miR-665. CircRNF121 and miR-665 were enriched in the Ago2 pellet relative to the control IgG immunoprecipitate, and endogenous circRNF121 pulldown was specifically enriched in miR-665-transfected cells (Fig. 4g). FISH experiment further confirmed the colocalization of circRNF121 and miR-665 (Fig. 4h).

Through bioinformatics analysis, we hypothesized that MYD88 might be a direct target of miR-665 (Fig. 4i). The direct interaction was confirmed by a dual-luciferase reporter assay (Fig. 4j). As shown in Fig. 4k, l, MYD88 expression was significantly increased after overexpression of circRNF121, and this promotion was reversed by co-transfection with miR-665 mimic. In contrast, sicirc-2 inhibited MYD88 level after transfection, and this effect was reversed by co-transfection with an inhibitor of miR-665. These results confirmed that circRNF121 sponged miR-665, and indirectly regulated MYD88 expression.

### CircRNF121/miR-665/MYD88 axis mediates ECM degradation, proliferation, and apoptosis in OA progression

**Fig. 5:**
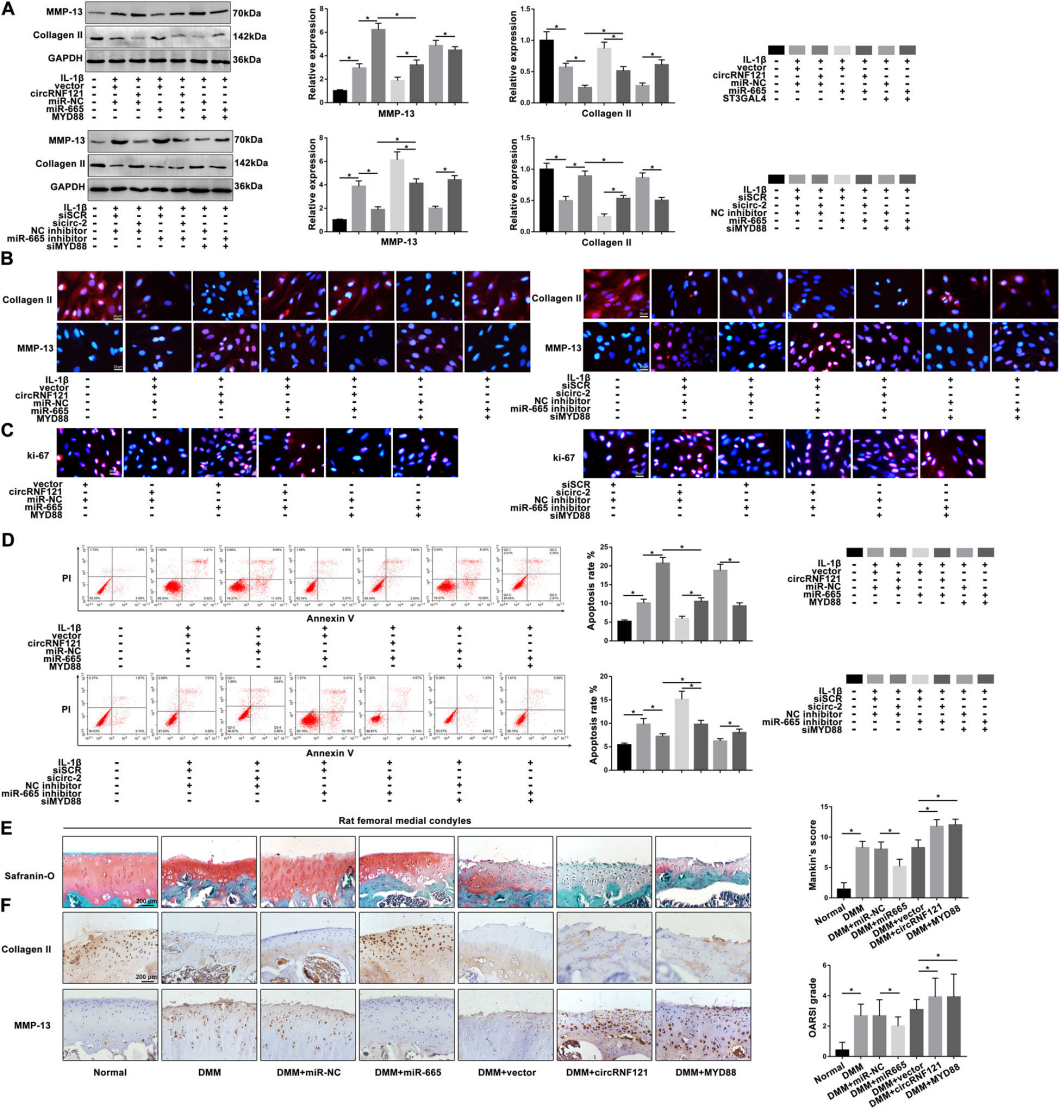
CircRNF121/miR-665/MYD88 axis mediates ECM degradation, proliferation, and apoptosis in OA progression. **a** MMP-13 and collagen II were detected by western blot. **b** Expression of MMP-13 and collagen II was presented by immunofluorescence staining. **c** The ki-67 intensity was shown by immunofluorescence staining. **d** The apoptotic rates of treated chondrocytes were measured by flow cytometry. Viable and nonviable apoptotic cells are regarded as apoptotic cells. **e** Histologic section of cartilage structures of SD rats was stained by safranin-O in each group. **f** The MMP-13 and collagen II levels in vivo were measured by IHC. Data were means ± SD of three independent assays (*P < 0.05).

Based on the above results, we performed rescue experiments to explore the function of the circRNF121/miR-665/MYD88 axis in the OA progression. As shown in Fig. 5a, b, overexpression of circRNF121 and MYD88 promoted ECM degradation. Interestingly, overexpression of miR-665 reversed this effect and inhibited IL-1β-induced ECM degradation. Conversely, the level of MMP-13 in chondrocytes knocked down by circRNF121 or MYD88 was reduced, whereas inhibition of miR-665 effectively blocked this effect. We next hypothesized that the circRNF121/miR-665/MYD88 axis also mediated the bioactive function of chondrocytes. As shown in Fig. 5c, d, miR-665 effectively reversed the circRNF121/MYD88-mediated antiproliferative and apoptotic effects. In contrast, sicirc-2/siMYD88-mediated proliferative and antiapoptotic effects were abrogated by miR-665 inhibitor.

The DMM model was established in vivo to evaluate the regulation role of circRNF121/miR-665/MYD88 axis during OA. Four weeks after DMM surgery, we injected various reagents into the knee joint of SD rats for treatment. The medial femoral condyles of models were harvested and analyzed 4 weeks after the injection. As shown in Fig. 5e, the complete organizational structure was shown in the miR-665 group compared with the miR-NC group by safranin-O staining. Overexpressed circRNF121/MYD88 group indicated severe damage to the superficial layer, which promoted the severity of OA compared with the vector group. Furthermore, miR-665 could effectively reverse the loss of collagen II in the DMM model, while inhibiting the expression of MMP-13. Overexpressed circRNF121/MYD88 regulated collagen II and MMP-13 levels in OA progression (Fig. 5f). These data illustrated that the circRNF121/miR-665/MYD88 axis mediated ECM degradation, proliferation, and apoptosis in OA progression, and provided promising therapeutic targets for the rehabilitation of OA.

### MYD88 promotes the activation of NF-кB pathway

**Fig. 6:**
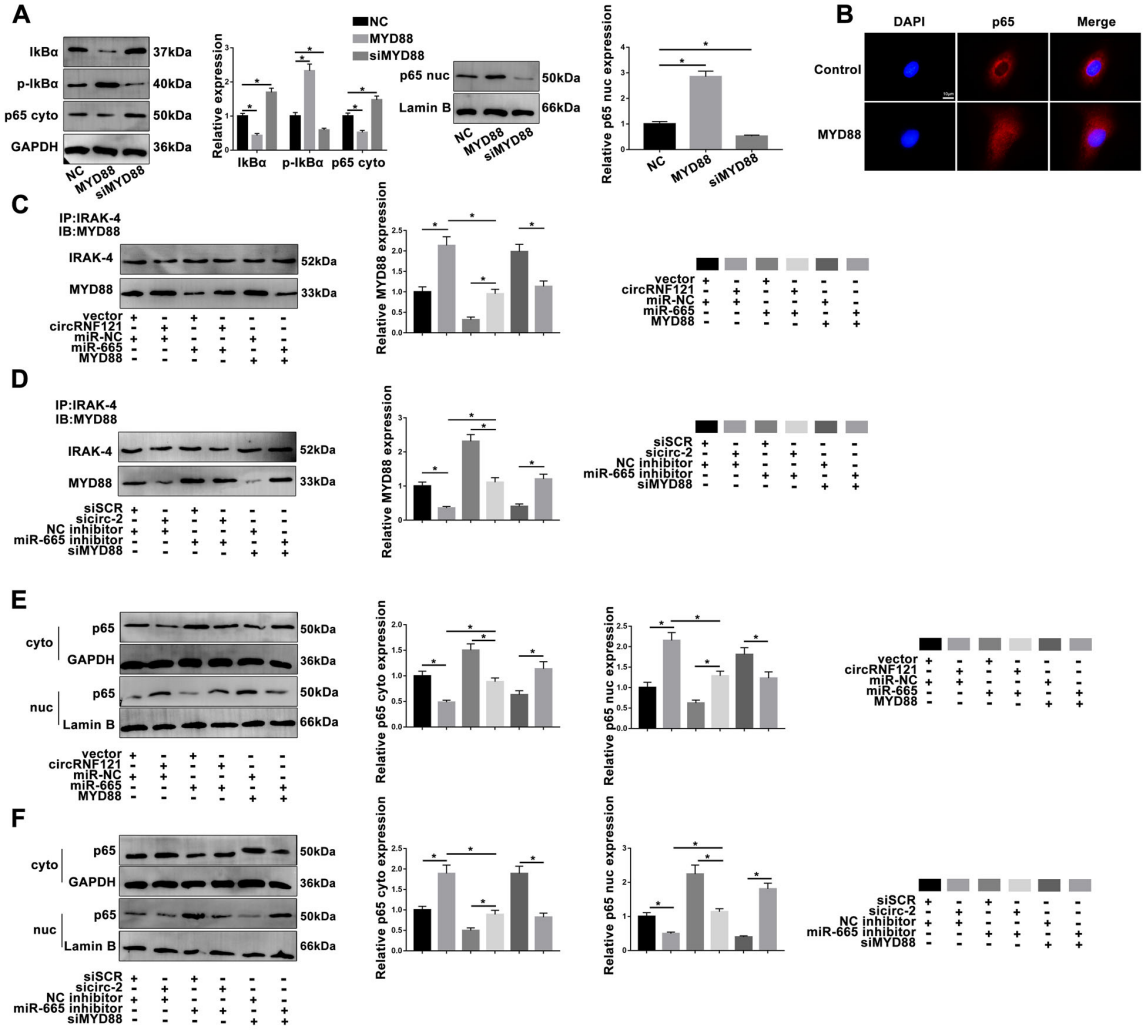
Altered MYD88 promotes the activation of NF-кB signaling pathway. **a**, **b** The effect of MYD88 on NF-кB pathway was detected by western blot and immunofluorescence staining. **c**, **d** CircRNF121 and miR-665 impacted the IRAK-4-binding MYD88 expression. **e**, **f** The effect of circRNF121/miR-665/MYD88 axis on NF-кB signaling pathway was detected by western blot. Data were means ± SD of three independent assays (*P < 0.05).

To further explore the molecular mechanism of the circRNF121/miR-665/MYD88 axis during OA progression, the NF-кB pathway was analyzed. MYD88 promoted phosphorylation of IkBα, which contributed to p65 entry into the nucleus, and ultimately facilitates the activation of NF-кB signaling pathway (Fig. 6a, b). By co-IP assay, the combination of MYD88 and IRAK-4 was modulated by circRNF121 and miR-665 (Fig. 6c, d). In order to announce the regulation of the NF-кB pathway by circRNF121 and miR-665 during OA progression, rescue experiments were further performed. As shown in Fig. 6e, f in the chondrocytes transfected with circRNF121, p65 was activated and entered the nucleus. However, these effects were effectively rescued in chondrocytes co-transfected with miR-665. Sicirc-2-mediated inhibition of NF-кB was abolished by miR-665 inhibitor. These data suggested that the circRNF121/miR-665/MYD88 axis mediated the activation of NF-кB pathway.

### Molecular mechanism of LEF1 and circRNF121/miR-665/MYD88 axis in chondrocytes

**Fig. 7:**
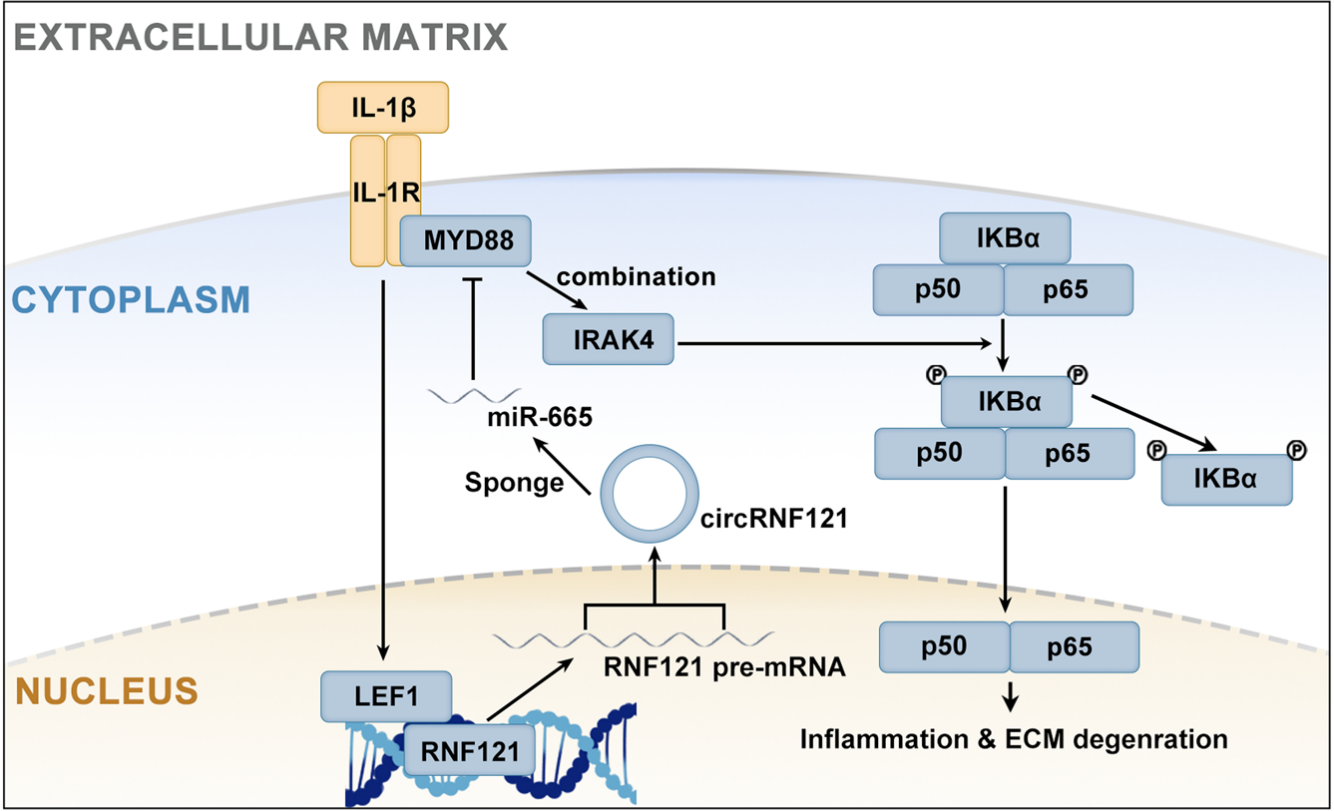
Molecular mechanisms of LEF1 and circRNF121/miR-665/MYD88 axis in chondrocytes. The molecular mechanisms of the current study.

Based on the above findings, the molecular mechanism of the current study is shown in Fig. 7. First, IL-1β promoted the expression of the transcription factor LEF1. LEF1 bound to the promoter region of RNF121 and activated transcription. The exon regions of the RNF121 pre-mRNA were back-spliced to form circRNF121. CircRNF121 sponged miR-665 that inhibited the expression of MYD88 through ceRNA. The circRNF121/miR-665/MYD88 axis mediated the activity of the NF-kB pathway by regulating the combining of MYD88 to IRAK-4.

## Discussion

Due to the self-regeneration potential of AC and limited migration ability, the damaged AC could not be repaired by itself, and eventually develops into OA. To develop effective therapeutics, it was important to investigate the underlying mechanisms of OA progression. This study provided an in-depth understanding of circRNF121/miR-665/MYD88 crosstalk that was regulated by LEF1 during OA progression via NF-кB signaling pathway.

The essential role of LEF1 in osteoarthritis was demonstrated in the previous study^27,28^. In the subsequent study, altered LEF1 expression was discovered by the sample matrix and further confirmed by q-PCR in tissue samples. In chondrocytes treated with IL-1β, the results of CHIP assay showed that LEF1 bound the RNF121 promoter. However, 35 AC tissues were analyzed and showed that the expression level of RNF121 was not dramatically correlated with OA. The expression of RNF121 was not increased in LEF1-overexpressing chondrocytes. Therefore, LEF1 promoted RNF121 mRNA transcription but not translation. The emphasis of this research was further explored on the circRNAs of RNF121.

Abundant studies have shown that abnormal circRNA expression was a critical issue in the development of OA. Altered expression of circRNA played an important role in the process of OA^29–31^. Zhao et al. demonstrated that Circ_0136474 and MMP‐13 suppressed cell proliferation, while it enhanced cell apoptosis by competitive binding to miR-127-5p in OA^32^. Four circRNAs associated with RNF121 were selected and analyzed in the current study. Together, the present findings confirmed that aberrant expression of circRNF121 was closely related to OA progression. In LEF1-overexpressing chondrocytes, the expression level of circRNF121 was significantly increased. Functionally, circRNF121 promoted the degradation of chondrocyte ECM, inhibited the proliferation of chondrocytes, and induced apoptosis. These proinflammatory effects were reversed after knocking down circRNF121. Although these results confirmed the involvement of circRNF121 in OA, the exact mechanism mediated by circRNF121 during OA progression remained unclear.

CeRNA theory played a role in the underlying mechanisms. MiRNAs could cause gene silencing by binding to mRNA. At the same time, circRNA could regulate gene expression by competitively binding to miRNA, thereby releasing the inhibitory effect on target mRNA. Recent literature pointed out the possible correlation between CircRNA VMA21 and miR-200c^33^. In our study, MYD88 and circRNF121 were identified as targets for miR-665. This conjecture confirmed that there was indeed an interaction between circRNF121 and miR-665. Modulation of miR-665 and circRNF121 mediated MYD88 expression. Functionally, upregulation of circRNF121 promoted OA progression, while upregulation of miR-665 could effectively reverse this regulation. Furthermore, co-transfection of circRNF121 and miR-665 mediated the biological activity of chondrocytes. MiR-665 was found to be involved in the development of OA by inhibiting circRNF121 function. The inhibitory effect of miR-665 on the DMM model was confirmed in vivo. Conversely, circRNF121 and MYD88 upregulated MMP-13 level, and then promoted the destruction of cartilage tissue in the DMM model. Comprehensive data provided enhance the evidence that the circRNF121/miR-665/MYD88 axis affected the OA progression.

The NF-кB pathway promoted the expression of OA-related proteins, such as RUNX2 and VEGF^17,18,20,34^. Many studies have confirmed that MYD88 played an important role in the OA process in recent years^35–39^. Here, modulation of miR-665 and circRNF121 resulted in altered MYD88 expression. MYD88 promoted the entry of p65 into the nucleus of the NF-кB pathway, thereby promoting the progression of OA. Altered expression of circRNF121 and miR-665 affected the binding of IRAK-4 to MYD88, which could promote phosphorylation of IKBα and activity of NF-кB pathway. Co-transfection of circRNF121 and miR-665 also regulates molecules involved in NF-кB pathway.

In conclusion, we reveal the essential role of circRNF121/miR-665/MYD88 axis that was modified by LEF1 via NF-кB pathway during OA progression. However, OA was still a multifactorial disease, and more research was required to delve into other molecular mechanisms. Current research provided valuable information about the therapeutic targets of OA.

## Supplementary information

Supplementary Figure

Supplementary Table S1

Supplementary Table S2

Supplementary Figure and Table legend
