# Correction: LEF1 mediates osteoarthritis progression through circRNF121/miR-665/MYD88 axis via NF-кB signaling pathway

**DOI:** 10.1038/s41419-020-02852-9

**Published:** 2020-08-11

**Authors:** Tianfu Wang, Zhiyu Hao, Changcheng Liu, Lebin Yuan, Li Li, Menghong Yin, Qing Li, Zhiming Qi, Zi Wang

**Affiliations:** 1 Department of Sports Medicine, Dalian Municipal Central Hospital, Dalian, 116033 Liaoning Province China; 2 Department of Spinal Surgery, The Second Hospital of Dalian Medical University, Dalian, 116033 Liaoning Province China; 3 Department of Medical Imageology, Dalian Medical University, Dalian, 116044 Liaoning Province China

Correction to: *Cell Death and Disease*

10.1038/s41419-020-02769-3 published online 30 July 2020

The original version of this Article contained errors in the author affiliations.

Affiliations incorrectly read as follows:

1. Department of Sports Medicine, Dalian Municipal Central Hospital, Dalian 116033, Liaoning Province, China.

2. Department of Spinal Surgery, The Second Hospital of Dalian Medical University, Dalian 116033, Liaoning Province, China.

3. Department of Medical Imageology, Dalian Medical University, Dalian 116044, Liaoning Province, China.

This has now been corrected in both the PDF and HTML versions of the Article.

