# Supplementary figures and images for: LEF1 mediates osteoarthritis progression through circRNF121/miR-665/MYD88 axis via NF-кB signaling pathway
Source: Cell Death Dis. 2020 Jul 30;11(7):598. doi: 10.1038/s41419-020-02769-3 (PMC7393488; doi:10.1038/s41419-020-02769-3)

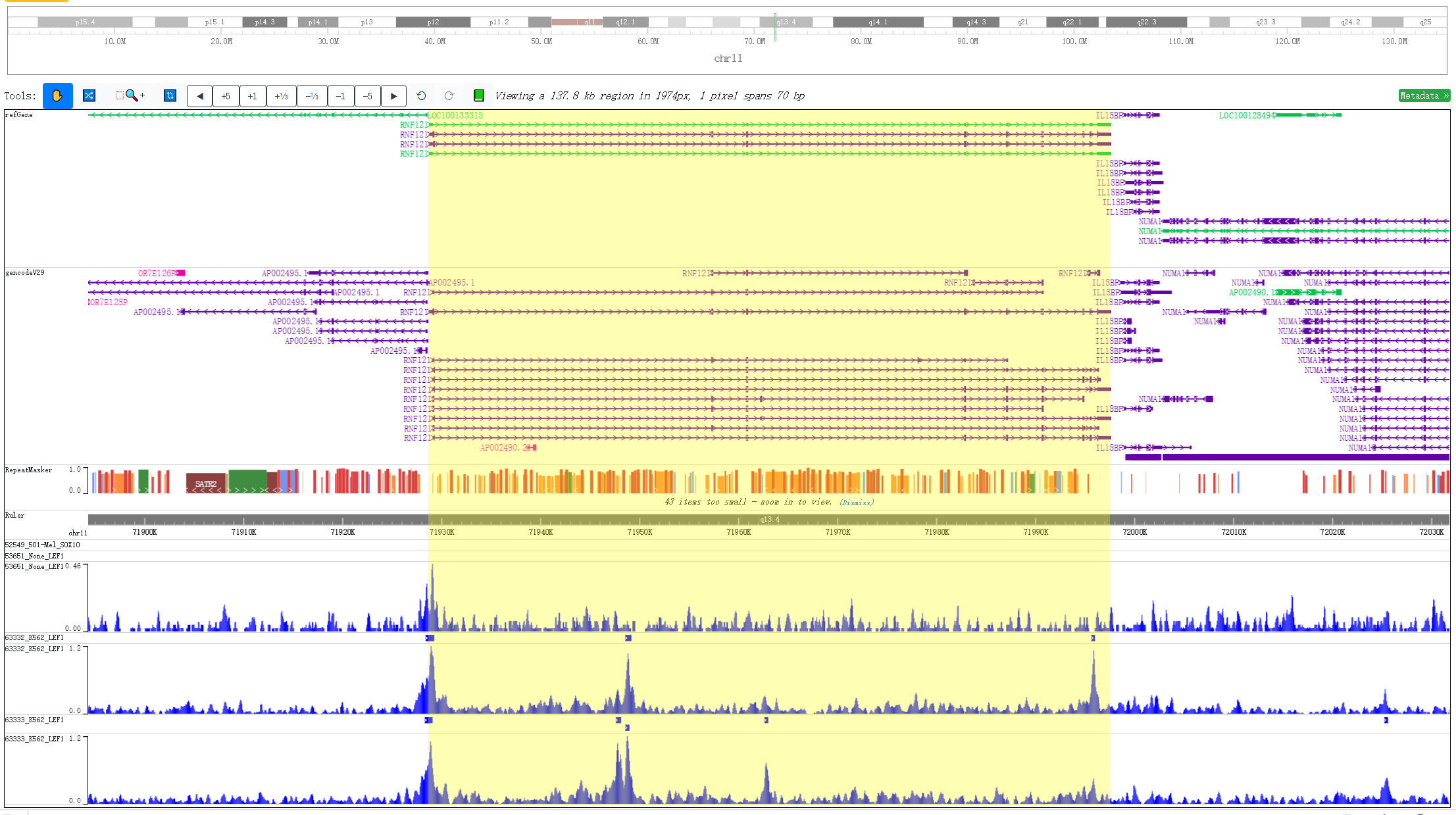

Supplement: Supplementary file 1 — Supplementary Figure [file 41419_2020_2769_MOESM1_ESM.png]
