# Supplementary material for: LEF1 mediates osteoarthritis progression through circRNF121/miR-665/MYD88 axis via NF-кB signaling pathway
Source: Cell Death Dis. 2020 Jul 30;11(7):598. doi: 10.1038/s41419-020-02769-3 (PMC7393488; doi:10.1038/s41419-020-02769-3)
Supplement: Supplementary file 4 — Supplementary Figure and Table legend [file 41419_2020_2769_MOESM4_ESM.docx]

**Supplemental Figure S1 Analysis results Chip-seq data of LEF1.**

LEF1 was predicted to combine with the promoter region of RNF121 according to the high-quality Chip-seq data in the Cistrome Data Browser.

**Supplemental Table S1 Standardized mRNA expression matrix.**

Microarray data of mRNAs was collected from GEO datasets (GSE114007) via the GPL11154 platform. The mRNA expression matrix was normalized using a robust multi-array averaging method with ‘affy’ and ‘simpleaffy’ packages of ‘R’ software. LogFC, log fold change; AveExpr, average expression.

**Supplemental Table S2 The relevant primers of current study.**

CircRNF121, Hsa_circ_0023404, circle RNA [ring finger protein 121](https://www.ncbi.nlm.nih.gov/gene/55298); LEF1, Lymphoid enhancer-binding factor 1; GAPDH, glyceraldehyde 3-phosphate dehydrogenase; U6, U6 small nuclear RNA.
